# Evaluating the Inappropriate Prescribing and Utilization of Caspofungin, a Four-Year Analysis at a Teaching Hospital in Saudi Arabia

**DOI:** 10.3390/antibiotics10121498

**Published:** 2021-12-07

**Authors:** Abdulwahab Aldrees, Leen Ghonem, Fahad Almajid, Mazin Barry, Ahmed Mayet, Abdulellah M. Almohaya

**Affiliations:** 1 Infectious Disease Unit, College of Medicine and King Saud University Medical City, King Saud University, Riyadh 12372, Saudi Arabia; abdaldrees@ksu.edu.sa (A.A.); almajid@ksu.edu.sa (F.A.); mbarry@ksu.edu.sa (M.B.); 2 Department of Medicine, Al-Diriyah General Hospital, Ar-Rihab, Ad Diriyah 13717, Saudi Arabia; 3 Department of Pharmacy, King Saudi University Medical City, Riyadh 11451, Saudi Arabia; lghonem@ksu.edu.sa (L.G.); iymayet@gmail.com (A.M.)

**Keywords:** antifungal, prescription, critical care, utilization, Caspofungin

## Abstract

The appropriate use of antimicrobial agents improves clinical outcomes and reduces antimicrobial resistance. Nevertheless, data on inappropriate prescription and negative outcomes are inconsistent. The objective of this study was to assess the prescription appropriateness of Caspofungin at a tertiary teaching hospital in Saudi Arabia and the impact on mortality at 30 days. A retrospective chart review was performed for patients who received Caspofungin from May 2015 to December 2019 to obtain prescription information and culture and susceptibility tests. The appropriateness of the dosage (ApD), initiation time (ApI), agent selection (ApS), and duration of therapy (ApDUR) was evaluated based on recommendations of the infectious diseases society of America. 355 eligible patients who received 3458 Caspofungin doses were identified. Their median age was 54 years (range 18–96). Overall, 270 (76.1%) patients received empirical prescriptions, of which 74.4% had the appropriate dose, and 56.3% had received it for more than five days, despite no proven Candida infection. This was not influenced by past medical history (*p* = 0.394). Only 39% of patients who received definitive prescriptions met all four study criteria for appropriate prescription. Therefore, antimicrobial stewardship programs can improve the appropriate utilization of antifungal therapies.

## 1. Introduction

Invasive fungal infections (IFIs) result in high morbidity and mortality [1]. In tertiary care hospitals, healthcare-associated bloodstream infections are most commonly caused by Candida [2]. However, the inappropriate use of antifungal therapy has resulted in the emergence of resistance [3]. Non-albicans Candida species, which frequently show resistance to fluconazole, are increasingly recognized as the cause of invasive fungal infections [4] In 2014, Omrani et al. from Saudi Arabia reported invasive bloodstream Candida infections at a median rate of 1.65 per 1000 hospital discharges per year [5,6].

Caspofungin, an echinocandin antifungal agent, has been approved by the United States Food and Drug Administration (US-FDA) for candidemia, esophageal candidiasis, and invasive Candida infections (ICI) that are refractory or intolerant to other agents. It is also prescribed as empiric therapy when invasive fungal infection is suspected. According to the manufacturer, the dose adjustment is 35 mg in patients with moderate hepatic impairment with a Child-Pugh score of ≥7 points. However, adherence to this recommendation is unknown [7].

Reports indicate that more than 50% of antimicrobials are inappropriately prescribed [8], more than that for antifungal agents, which ranges between 45–70% [9,10]. The absence of a widely accepted template for antifungal prescription appropriateness may be a reason for this discrepancy. Antimicrobial stewardship (ASP) interventions for antifungal therapy are recommended [11] to avoid adverse side effects, prevent resistance, reduce the length of hospital stay, and improve cost effectiveness [12]. However, data on antifungal agent utilization are lacking, especially in the Middle East. Thus, a periodic evaluation and reporting of antifungal agents’ prescriptions and their appropriateness can improve physicians’ prescribing practices. Although there are insufficient guidelines on assessing antibiotic prescriptions’ appropriateness, most of the studies focus on the following domains: early initiation, correct dose, timely modification to the regimen, and appropriate duration [13]. In this institute, Caspofungin has been part of the institute formulary for more than 10 years but we recently observed an increase in Caspofungin prescribing. As such, the main objective of this study was to explore the rate and outcome of inappropriate prescribing of Caspofungin in an academic tertiary care center in Riyadh, Saudi Arabia using these domains. To the best of our knowledge, this is the first study in the region that looks at antifungal agent use and its relationship to mortality.

## 2. Results

### 2.1. Patients

Four hundred and ninety-three patients had received Caspofungin doses over the study period. A total of 138/493 (28%) patients were excluded because they either received only a single dose, had incomplete investigations, or died very early during therapy. Thereafter, 355 unique patients who received a total of 3458 cumulative doses of Caspofungin were included in the study. The patients were divided into two groups, the empiric therapy (ET) group (*n* = 270, 76.1%) and the definitive therapy (DT) group (*n* = 85, 23.9%). Overall, the median patient age was 54 years (range 18–96 years), with patients in the ET group slightly younger than those in the DT group (median age, 53 vs. 56 years). The males and females were equally distributed in the entire cohort. Within this cohort, 57.2% of the patients (*n* = 203) were in the intensive care unit (ICU) and were predominantly in the ET group rather than in the DT group (60% vs. 48.2%). Only 16.1% of the entire cohort had a high Child-Pugh score (≥7 points) (*n* = 57), Table 1. 

### 2.2. Indications

Most of the patients (93%) in the ET group had at least one risk factor for IFI, while the remaining 19 patients were prescribed empiric Caspofungin in the absence of any risk factor. The identified risk factors for IFI were the presence of a central venous catheter (CVC) in two-thirds of the patients (*n* = 161, 59.9%) or a recent intra-abdominal surgery (IAS) in 44.8% (*n* = 121) of the patients (Table 2). In the DT group, most of the cases were treated with Caspofungin for either candidemia (*n* = 39, 45.9%) or ICI (*n* = 38, 44.7%). The remaining nine patients were diagnosed with either esophageal candidiasis (*n* = 5, 5.9%) or invasive aspergillosis (*n* = 3, 3.5%). All cases with candidemia had undergone a transthoracic echocardiogram and had no documented endocarditis. The most common species of Candida were Candida albicans (*n* = 29, 34.5%), Candida glabrata (*n* = 22, 25.3%), and Candida tropicalis (*n* = 10, 11.5%), Table 3.

### 2.3. Caspofungin Duration and Doses

Most of the patients received less than 14 days of Caspofungin (79.7%), with a median duration of 7 days. A duration longer than 14 days was significantly more in the DT group compared to the ET group (36.5% vs. 15.2%, *p* < 0.001, odds ratio = 3.2, 95%CI: 1.85–5.57). Nevertheless, the ET group had higher cumulative doses compared to the DT group (2279 vs. 1179 doses). Among the ET group, half of the patients received empiric anti-Candida therapy for longer than 5 days (152/270, 56.3%), resulting in an unnecessary 1131 doses. This was more among ICU patients (60.5%) than those in general care (39.5%). In the DT group, the median duration of Caspofungin was similar for patients with ICI and candidemia at 14 and 13 days, respectively. The majority of the cohort had received the correct loading dose (92.4%, *n* = 328), maintenance dose (80.6%, *n* = 286), or both (73.5%, *n* = 261) with no statistical differences between the ET and DT groups.

### 2.4. Appropriateness Evaluation in the Definitive Therapy Group

In our study, the appropriateness of Caspofungin prescriptions ranged from 70.6% to 82.4%, with the highest being the appropriate duration (ApDUR) (82.4%), followed by the appropriate agent selection (ApS) (76.5%), appropriate initiation time (ApI) (75.3%), and appropriate dose (ApD) (70.6%). The four criteria were present in 33 patients (38.8%), while 71.8% had three or two criteria (42.4% and 29.4%, respectively), and only one patient, who had esophageal candidiasis, did not fit any of the appropriateness criteria. Prescriptions with inappropriate doses had a statistically significant association with the rate of mortality at 30 days (53% vs. 24%, *p* = 0.02). There was no association between inappropriate dose and past medical history among patients with available history (*p*-value= 0.394).

### 2.5. Inpatient All-Cause Mortality at 30-Days

Among the entire cohort, a statistically significant association was observed between 30-day mortality and the following: patients aged 65 years and older (odds ratio, OR = 3.2), patients admitted to the ICU at the time of antifungal initiation (OR = 4.6), and patients with hepatic dysfunction defined as a Child-Pugh score ≥ 7 points (OR = 7.4). Among the four appropriateness categories studied, those who received inappropriate dosing of Caspofungin had a higher mortality (OR = 2.5), Table 4.

## 3. Discussion

In this retrospective study, compared to previous reports, there was a higher inappropriate utilization of Caspofungin, especially in ICU [14,15]. Such inappropriate practices in prescribing might lead not only to increased mortality, as shown in this study, but could also result in evolving antifungal resistance which is underestimated worldwide [16].

The most common risk factor for prescribing ET was the presence of CVC (59.9%) and recent IAS (44.8%). Both CVC and IAS are known to introduce Candida species into sterile body sites as a result of skin and gastrointestinal barrier disruption [14,17]. Due to the risk of high mortality from ICI [18], early initiation of empirical antifungal therapy is justified, although not yet standardized. Some physicians justify the need for empirical antifungal therapy by calculating the Candida score established by Leon et al. [19]. Nevertheless, it has not been endorsed or approved by the Infectious Diseases Society of America (IDSA) guidelines. In our study, more than half of the ET cases (51.4%) were not justified because of the absence of risk factors or the presence of a single risk factor for ICI. Even with the application of the Candida score, only 159 patients (42.2%) had a positive score of > 2.5, which indicates a lower proportion of those at high risk of IFI. 

Notably, in our cohort, a larger proportion of patients in the ET group were among those who died shortly after (85% of deaths) or were in the ICU on the first day of Caspofungin initiation (79% of ICU patients). More than half (56.3%) of the ET group continued beyond five days from initiation, even though there was no evidence of ICI infection. Consequently, 1131 unnecessary Caspofungin doses were continued among approximately two-thirds of the patients in the intensive care unit. To reduce wastage, the implementation of an antibiotic stewardship program on Caspofungin (antifungal utilization) in the critical care unit is needed.

In the definitive group, using our criteria of appropriateness, our center scored highest in providing the appropriate duration but lowest in providing the appropriate dosage of Caspofungin. In our study, 61.2% of the Caspofungin doses in the DT group failed to satisfy the four criteria of appropriateness. Although there was no significant association, the inappropriate prescriptions were higher among patients older than 65 years and in patients with candidemia and lower among patients in the ICU. There were no cases of endocarditis in this cohort. Inappropriate dose (but not overall appropriateness) was also found to be an independent risk factor that was twice as likely to result in death at 30 days in the definitive group, irrespective of age and the patient’s ICU status. Although previous studies have used similar definitions to this study, there is no consensus on the definition of appropriate prescriptions. Thus, there is great variability in the appropriateness of prescriptions between different definitions and different patient populations. For example, antifungal inappropriateness reached 45% in a study performed in a transplant center in Spain [9] whereas, in a non-transplant tertiary center in Thailand, antifungal inappropriateness was as high as 70% (42/57) [10]. This may obscure further comparative analysis. 

One in every four prescriptions in this cohort was inappropriately dosed, resulting in a significantly higher death rate at 30 days (*p* = 0.01). For Caspofungin prescriptions, 82.4% lacked any of the four criteria of prescription appropriateness used in this study. A number of other studies reported an incorrect dosing of Caspofungin from 16% to 38% of all antifungal prescriptions [14,20]. These results were comparable to the current study findings. Ninety-four incorrect doses (26.5%) of Caspofungin were administered, of which there were 67 maintenance doses (71.3%). This was primarily due to the unadjusted dose for high Child-Pugh scores. There have been concerns with adjusting the Caspofungin dose in cirrhotic patients based on the Child-Pugh score, which can lead to sub-therapeutic concentrations of the drug in blood and tissues [21,22]; however, the manufacturer’s recommendation has not been revised. As previously mentioned, more incorrect dosages were prescribed in critical care than in non-critical care patients (28.6% vs. 23.7%), which resulted in a higher all-cause mortality (39% vs. 20.3%, *p* = 0.01). It is not clear whether these mortalities were among patients who were already at a higher risk of death or as a result of sub-therapeutic antifungal therapy. Therefore, physicians’ awareness and education on Caspofungin dosing, particularly in ICU settings, are warranted. 

As in the case of antibacterial agents, limited options are available to combat resistant fungal infections. Unnecessary and prolonged exposure to echinocandins have resulted in the emergence of echinocandins resistant Candida species [23]. It is currently known that the appropriate prescribing and monitoring of antifungal agents could result in cost reduction by 37%, and, importantly, a reduction of the burden of resistance to antifungal agents [16,24].

Delay in starting antifungal therapy after confirming the diagnosis of ICI has been linked to a twofold increase in the risk of death [25]. Conversely, in the current study, there was a higher mortality among those who started early antifungal therapy with no statistical association. This may be because of the pre-existing critical illnesses in the early initiation group (*n* = 13) compared to non-critical cases (*n* = 4), as well as the all-cause mortality principle that was employed. Such high mortality risk can be reduced by implementing effective stewardship programs [9].

This study had a few limitations. First, this was a single-center and retrospective study which limits the generalization of the conclusion. Second, other coexisting confounding mortality risk factors were not considered. Third, drug–drug interaction and intravenous to oral switch as markers for prescriptions appropriateness were not evaluated. However, despite these limitations, this study highlights important prescribing information on Caspofungin. 

## 4. Materials and Methods

### 4.1. Study Design, Settings, and Data Collection

A retrospective cohort study at an academic tertiary care center in Riyadh, Saudi Arabia to assess the appropriate utilization of Caspofungin was conducted. All consecutive hospitalized patients aged 18-years-old and above who received Caspofungin from May 2015 to December 2019 were screened for inclusion in the study. Patients who received only a loading dose were excluded, aiming for strict evaluation of both loading and maintenance doses. Patients who died within 48 h of therapy initiation or had no investigations to evaluate the appropriate duration of therapy (i.e., repeated cultures for candidemia or radiological investigations for invasive candidiasis) were also excluded.

The institution is a 1200-bed referral tertiary teaching hospital in Saudi Arabia that runs a reference laboratory for microbiology. In accordance with the latest Clinical & Laboratory Standards Institute (CLSI) standards, Candida species from blood specimens were identified using the VITEK^®^ 2 healthcare system (bioMérieux, Inc., Hazelwood, MO, USA) and susceptibility testing was performed using bioMérieux VITEK^®^ 2 Fungal Susceptibility (AST-Y07). In this institute, Caspofungin prescription is restricted to infectious disease specialists as part of the hospital’s antimicrobial stewardship program. This is to ensure the appropriate utilization of antimicrobials, improve safety outcomes, and reduce microbial resistance. 

Patient demographics from electronic medical records, including age, sex, site of care on the day of therapy initiation, and history of chronic illnesses were obtained from medical records. Patient’s risk factors for fungal infection, defined as the presence of any of the following: an indwelling CVC, recent IAS within the last three months, admission to ICU, continuous renal replacement therapy, total parenteral nutrition, corticosteroid therapy, or use of immunosuppressive agents, were also collected. The relevant laboratory data, including complete blood count, renal and liver function tests, and coagulation profiles were documented. Descriptions of the patient’s fungal disease, including the indication for antifungal prescription, clinical and radiological findings, microbiological and histopathological findings, culture and susceptibility tests, and serological test results (i.e., Aspergillus galactomannan) were collected along with details of the Caspofungin therapy regimen (dosage and duration of therapy).

### 4.2. Study Definitions

Caspofungin therapies were classified into empiric therapy (ET) and definitive therapy (DT). For neutropenic patients, ET was defined as the use of Caspofungin to treat patients with persistent fever despite receiving broad-spectrum antibacterial therapy, without positive microbiological culture results. In non-neutropenic patients, ET was defined as therapy in critically ill patients who received initial treatment if they were febrile with any of the risk factors for invasive fungal infection, in the absence of any other known cause of fever. DT was defined as the use of Caspofungin to treat a documented fungal infection based on microbiological evidence with susceptibility testing. The definitions of candidemia and ICI were adopted from the IDSA guidelines [1].

Guided by the recommendations of the IDSA guidelines [1], the appropriateness (Ap) of Caspofungin use was evaluated using criteria adopted from a previous study [9]. This was applied by both a senior infectious disease specialist and an infectious disease clinical pharmacist. The criteria included appropriate dosage (ApD), appropriate initiation time (ApI), appropriate agent selection (ApS), and appropriate duration of therapy (ApDUR). The current study did not include an assessment of drug–drug interaction because of insufficient data among the patients. Overall appropriateness (OApp) was considered if the Caspofungin prescription met all four criteria of appropriateness. Of note, the empiric therapy group was evaluated using only the ApD and ApDUR criteria, while the definitive therapy group was evaluated using all four.

ApD of Caspofungin was defined as a loading dose of 70 mg (except in esophageal candidiasis, where the loading dose was not needed) followed by a maintenance dose of 50 mg, administered daily. In a setting of moderate hepatic impairment, defined as a Child-Pugh score of ≥7 points, the Caspofungin dose was adjusted to 35 mg in compliance with the manufacturer’s instruction [7]. Doses above and below the manufacturer’s recommendations were considered inappropriate. The initiation time for Caspofungin was considered appropriate (ApI) if the first dose was administered within 24 h of definitive or preliminary microbiological evidence of IFI. For the ApS criteria, Caspofungin was deemed appropriate if it covered the suspected fungi and was the first-line treatment recommended by the guidelines. The ApDUR was evaluated based on the following indications: for candidemia [26], days of therapy after the first documented negative blood culture; for ICI, therapy was continued until either clinical or radiological resolution of infection was observed [1,26]; for esophageal candidiasis, there was a minimum of 14 days of therapy.

### 4.3. Study Outcome

The primary outcome of this study was the observed appropriateness of Caspofungin therapy. The secondary outcome was the association between the appropriateness of Caspofungin prescription and all-cause inpatient mortality at 30 days after the first dose of Caspofungin.

### 4.4. Statistical Analysis

Continuous variables were expressed using mean and standard deviation, while categorical variables were summarized as counts and percentages and were examined using the χ^2^ test. SPSS software (version-23, IBM Corp., Armonk, NY, USA) was used to perform the statistical analyses. Associations of appropriateness of Caspofungin therapy with all-cause mortality were tested using univariate and multivariate logistic regression to estimate the odds ratios (ORs), adjusted odds ratios (aORs), and 95% confidence intervals (CIs).

## 5. Conclusions

A large portion (three-quarters) of Caspofungin prescriptions were written empirically, mostly prescribed from the ICU. Approximately two-thirds of the definitive therapy prescriptions were not appropriate and incorrect dosing led to a higher mortality rate. Antimicrobial stewardship programs with special focus on antifungal utilization in the ICU may improve the appropriate use of such therapies. Further studies to explore other antifungal agents’ prescriptions and to test the effect of stewardship programs are recommended.

## Figures and Tables

**Table 1:** Demographic, site of care, dosing, and outcome among patients who received Caspofungin during the study period, stratified by the indication (*n* = 355).

Study Variables		Total	Indications	
		Overall, *n* = 355, (100%)	Empiric Therapy (ET) *n* = 270, (76.1%)	Definitive Therapy (DT) *n* = 85, (23.9%)
**Age** **Range 18–96**	median	*54 years*	*53 years*	*56 years*
	Age > 65 years	97 (27.3%)	68 (25.2%)	29 (34.1%)
	Age ≤ 65 years	258 (72.7%)	202 (74.8%)	56 (65.9%)
**Sex**	Female	173 (48.7%)	131 (48.5%)	42 (49.4%)
	Male	182 (51.3%)	139 (51.5%)	43 (50.6%)
**Child-** **Pugh Score**	Score ≥ 7 points	57 (16.1%)	44 (16.3%)	13 (15.3%)
	Score < 7 points	298 (83.9%)	226 (83.7%)	72 (84.7%)
**Site of care**	Critical Care	203 (57.2%)	162 (60%)	41 (48.2%)
	Ward	152 (42.8%)	108 (40%)	44 (51.8%)
**Duration of Therapy**	Median days	7 days	6 days	12 days
	Cumulative Doses	3458 doses	2279 doses	1179 doses
	Duration ≤ 14 days	283 (79.7%)	229 (84.8%)	54 (63.5%)
	Duration > 14 days	72 (20.3%)	41 (15.2%)	31 (36.5%)
Dosing *	Correct Loading Dosing	328 (92.4%)	252 (93.3%)	76 (89.4%)
	Correct Maintenance Dosing	286 (80.6%)	217 (80.4%)	69 (81.2%)
	Appropriate dose (ApD) **	261 (73.5%)	201 (74.4%)	60 (70.6%)
**Outcome** **at 30 days**	Death	118 (33.2%)	101 (37.4%)	17 (20%)
	Survived	237 (66.8%)	169 (62.6%)	68 (80%)

DT (definitive therapy) was defined as the use of Caspofungin to treat a microbiologically proven infection according to Infectious Diseases Society of America criteria, while empiric therapy (ET) was defined as the use of Caspofungin in suspected but not confirmed invasive fungal infection. * Loading or maintenance doses that were above or below the manufacturer recommendation were considered incorrect. ** Appropriate dose (ApD) is defined as a correct loading and maintenance doses with appropriate dose adjustment.

**Table 2:** Risk factors and duration of empiric prescriptions (ET) of Caspofungin among patients in this study (*n* = 270):.

Parameter		*n* (%)
Number of risk factors	One risk factor	120 (44.4)
	Two risk factors	91 (33.7)
	Three risk factors	35 (13)
	Four risk factors	5 (1.9)
	Empirical therapy with no risk factors	19/270 (7)
No single risk factor among ET group	Median duration (range)	4 (2–18) days
	Empirical therapy out of critical care units	10/19 patients
	Cumulative doses	117 doses
Category of risk factor	Central venous line	161 (59.9)
	Recent abdominal surgery within 90 days	121 (44.8)
	Solid malignancy	49 (18.1)
	Renal Replacement Therapy	40 (14.8)
	Hematologic malignancy	24 (8.9)
	Total parental therapy	23 (8.5)
	Corticosteroids equivalent to Prednisolone 20 mg daily	7 (2.6)
	Human Immunodeficiency virus	2 (0.7)
Duration	Five days or less.	118 (43.7)
	Extended beyond 5 days,	152 (56.3)
	Median duration	10.5 days
	Cumulative extra doses	1131 doses
	Received duration while in critical care units.	92/152 (60.5%)
	Ended up with death at 30 days	51/152 (33.6%)

ET: empiric therapy was defined as the use of Caspofungin in suspected but not confirmed invasive fungal infection.

**Table 3:** Diagnosis, microbiology, and course details for patients with definitive Caspofungin therapy (*n* = 85).

Parameter		*n* (%)
Diagnosis	Candidemia	39 (45.9)
	Invasive candidiasis infections (ICI)	38 (44.7)
	Esophageal candidiasis	5 (5.9)
	Proven invasive aspergillosis	3 (3.5)
Species	*C. Albicans*	29 (34.5)
	*C. Glabrata*	22 (25.3)
	*C. Tropicalis*	10 (11.5)
	*C. Krusei*	6 (6.9)
	*C. Parapsillosis*	5 (5.7)
	Other Candida	10 (12.6)
	Aspergillus species	3 (3.4)
Caspofungin course	Continued, not shifted to another agent	41/85 (50.6)
	De-escalation to fluconazole was possible	17/41
	De-escalation to fluconazole was not possible *	24/41
	Shifted to another agent	44/85 (49.4)
	Susceptibility guided	37/44
	To another echinocandin	4/44
	Side effects (AKI and hepatitis)	2/44
	Worsening sepsis	1/44
Appropriateness Criteria	Prescriptions met appropriate duration of therapy (ApDur)	70/85 (82.4)
	Prescriptions met appropriate agent selection (ApS)	65/85 (76.5)
	Prescriptions met appropriate initiation time (ApI)	64/85 (75.3)
	Prescriptions met Appropriate dosing (ApD)	60/85 (70.6)
	Prescriptions met all four criteria	33/85 (38.8)

Candidemia, invasive candida infection (ICI), esophageal candidiasis, and invasive aspergillosis are defined according to the Infectious Diseases Society of America 2016 and EORTC/MSGERC 2020. * Either fluconazole resistant/intermediate (*n* = 22), used as an adjunct in invasive aspergillosis (*n* = 1), or allergic to fluconazole (*n* = 1).

**Table 4:** Predictors of all-cause mortality at 30 days among patients who received Caspofungin included in this study, using univariate and multivariate regression analysis (*n* = 355).

Predictors	Univariate Analysis		Multivariate Analysis				
	*p*-Value (0.05)	Odds Ratio (95% CI)	Coeff (B)	Standard Error (SE)	Wald X2	*p*-Value (0.05)	Adjusted Odds Ratio (95% CI)
Age above 65 years	<0.001	3.2 (1.94–5.14)	1.044	0.279	13.965	<0.001	2.84 (1.64–4.92)
Female sex	0.572	-	-	-	-	-	-
Therapy duration shorter than 14 days	0.167	-	-	-	-	-	-
Child-Pugh score ≥ 7 points	<0.001	7.4 (3.9–13.86)	1.728	0.357	23.461	<0.001	5.63 (2.80–11.33)
Admission to Intensive care unit	<0.001	4.6 (2.75–7.71)	1.439	0.286	25.261	<0.001	4.22 (2.41–7.39)
Incorrect loading dose	0.199	-	-	-	-	-	-
Incorrect maintenance dose	<0.001	2.7 (1.59–4.65)	-	-	-	-	-
inappropriate dose	<0.001	2.5 (1.55–4.09)	0.606	0.290	4.372	0.037	1.83 (1.04–3.24)

## Data Availability

The data that support the findings of this study are available on request from the corresponding author [A.A.]. The data are not publicly available due to privacy sensitivity.
